# Differential diagnosis of pseudohypopyon and discussion of Extranodal natural killer/T-cell lymphoma presenting as hypopyon panuveitis

**DOI:** 10.1186/s12886-022-02527-3

**Published:** 2022-10-06

**Authors:** Cem Evereklioglu

**Affiliations:** Department of Ophthalmology, Division of Uvea-Behçet Unit, Erciyes University Medical Faculty, Kayseri, Turkey

**Keywords:** Color, Endophthalmitis, Hypopyon, Neoplastic, Precipitation, Pseudohypopyon, Sediment, Tumoral

## Background

Given the recent attention on the distinction of “hypopyon” from “pseudohypopyon” for an early diagnosis of sight-threatening ocular or systemic diseases, especially for neoplastic masquerading syndromes, the recent article of Sukon et al. published in BMC Ophthalmology is most timely [1]. It is furthermore a credit to the authors to share their detailed experience of an initial misdiagnosis, and such a case presentation could prevent erroneous management in the future. The authors presented a 43-year-old woman with 2 months of decreased vision in the left eye with hypopyon panuveitis. The patient was diagnosed initially with endogenous endophthalmitis but was irresponsive to both antimicrobial and high-dose prednisolone therapies. The final diagnosis was extranodal natural killer/T-cell lymphoma and chemotherapy was initiated, though progressive visual loss was encountered within 9 months as a result of phthisis bulbi. The article of the authors is most timely for the differential diagnosis of hypopyon and pseudohypopyon. However, I would like to make some important notifications and contributions to the ocular findings of this unique case, which the authors called “hypopyon”. In my view, there are numerous objective and subjective clues in the article for making a true definition and differential diagnosis of “hypopyon” from “pseudohypopyon”.

## Main text

*First:* the definition or the nomenclature of the anterior chamber collection was defined as “hypopyon” and such terminology does not seem to be correct. The term “hypopyon”, a layered meniscus formation in the anterior chamber of the eye, is an etiological definition, indicating an advanced exudative stage of an acute intraocular inflammation that rapidly responds to corticosteroids [2]. Therefore, when hypopyon originates from autoimmune non-infectious uveitis or by exogenous and endogenous microbial infections, it classically presents with severe perilimbal (circumcorneal) dusky red hyperaemia with a violaceous hue (ciliary injection or flush) along with conjunctival, episcleral and deep scleral vasodilatations. The term “warm hypopyon” is used for such a “hot disease”. If we carefully look at Fig. 1 in the presented case, the involved left eye of the woman is typically white and there are no signs of the above-mentioned acute anterior uveitis such as ciliary injection. In addition, the patient did not complain of ocular pain, lacrimation or photophobia, all of which are cardinal symptoms of acute uveitis. Taken together, all the abovementioned symptoms and findings along with a painless decline in her vision should have suggested a non-infective etiology for this unique case. In addition, sediments in microbial endophthalmitis cause “purulent and yellow-coloured hypopyon formation”, which results in pus formation typically heaped up centrally rather than at its edges. Such macroscopic signs to the naked eye should have alerted the authors during the examination against infective endophthalmitis.

On the other hand, the term “pseudohypopyon” is used for anterior chamber precipitations due to non-inflammatory “masquerade syndromes of malignancy”, which contain neoplastic cell collections without a microbial agent or pus (sterile) [3]. Strictly speaking, the term “tumoral or neoplastic pseudohypopyon” should be preferred for such a collection to define it appropriately. However, repetitive head shaking or chronicity of the disease may finally cause mild ciliary injection along with secondary uveitis due to tumoral cell irritation of the uveal tissues. In other words, a pseudohypopyon may sometimes acquire a secondary inflammatory characteristic after uveal hazard. Therefore, if the woman had had microbial endophthalmitis during the initial presentation, as the authors thought, there should have been a “consistency” between the inflammatory nature of the sediment in the anterior chamber and the corresponding inflammatory appearance of the globe (a “bloodshot eye”). Indeed, “uninjected white eye” should not be expected in a “hot disease”, namely endogenous microbial endophthalmitis.

*Second:* the distinction of “pseudohypopyon” from “hypopyon” is vital for an ophthalmologist since their treatments and prognoses are entirely different [4]. For this reason, the sediment’s characteristics, namely its shape and behaviour are very important. To avoid confusion among clinicians, a true definition or description of the sediment is important to perform a correct diagnosis. Therefore, my next question is, did the authors evaluate the mobility of the sediment? In other words, I wonder whether the sediment was shifting or not? It is known that the etiology of the anterior chamber precipitation affects the position and movement of the sediment at various speeds. Indeed, a positional change of the patient may cause the dispersion of the meniscus if the sediment contains a lower concentration of fibrinous exudate (like in tumoral pseudohypopyon) and completely dislocates into another part of the eye *within seconds* upon permanent leaning to one side. This kind of moving sediment into the direction of head tilting is called “mobile-” or “shifting-pseudohypopyon” and is one of the pathognomonic features of “neoplastic pseudohypopyon”. So, if the authors had performed a positional change with their patients, they would probably have seen the shifting nature of the sediment. In turn, all infectious (microbial endophthalmitis) and non-infectious inflammations (i.e. HLA-B27+ acute anterior uveitis) result in “immobile, non-shifting (plastic) hypopyon” that contains a high level of fibrinous exudates with or without microbial agents, respectively. Notably, there is only one exception to this rule; the hypopyon in ocular Behçet disease is also “shifting” in nature, but the sediment dislocates slowly *within 5–10 minutes* upon posture change, not *within seconds* as seen in “tumoral pseudohypopyon”.

*Third:* the colour of the precipitate and the appearance of the perilimbal area and the sclera may indicate a specific etiology, and as stated above “uninjected white (cold) eye” should not be expected in a “hot disease”, namely endogenous microbial endophthalmitis [5–7]. Although the colour of both “non-infectious hypopyon” and “pseudohypopyon” are generally “white” or “greyish-white” in appearance, “yellow hypopyon” is encountered in microbial panophthalmitis [3]. If we check Fig. 1 again, the pinkish “blood-tinged” nature of the anterior chamber sediment is seen, which again indicates spontaneous hyphaemia due to leukaemia or lymphoma [8]. Such a collection should be described as “blood-streaked pinkish pseudohypopyon”.

## Conclusions

The presented case does give clear initial clues that the sediment in the anterior chamber of the eye is likely to be non-inflammatory “masquerade syndromes of malignancy”, suggesting an appropriate terminology of “pseudohypopyon”. Taken together, the type of collection for the present woman may be named as descriptive as possible, e.g., *“a 43-year-old woman with unilateral, pinkish-white, (possibly) shifting, 2-mm cold macro-pseudohypopyon (in a white eye)”*, which may direct physicians away from hypopyon with an infectious origin, and rather to a potential list of “neoplastic pseudohypopyon". Therefore, if sediment in the anterior chamber is evaluated with its full characteristics (colour, mobility and shape), the ophthalmologist can make (or exclude) a systemic diagnosis on time, predict the prognosis, and direct the selected patients to the proper physician for immediate medical or surgical treatment.

## Data Availability

Data sharing is not applicable to this correspondence as no datasets were generated or analysed during the current study.

## Abbreviation

HLA: Human leukocyte antigen
